# Transdifferentiation-Induced Neural Stem Cells Promote Recovery of Middle Cerebral Artery Stroke Rats

**DOI:** 10.1371/journal.pone.0137211

**Published:** 2015-09-09

**Authors:** Hui Yao, Mou Gao, Jianhua Ma, Maoying Zhang, Shaowu Li, Bingshan Wu, Xiaohu Nie, Jiao Jiao, Hao Zhao, Shanshan Wang, Yuanyuan Yang, Yesen Zhang, Yilin Sun, Max S. Wicha, Alfred E. Chang, Shaorong Gao, Qiao Li, Ruxiang Xu

**Affiliations:** 1 Affiliated Bayi Brain Hospital, General Hospital of Beijing Military Region, P.L.A, Beijing, 100700, PR China; 2 Neurosurgery Institute of Beijing Military Region, Beijing, 100700, PR China; 3 The Third Military Medical University, Chongqing, 400038, PR China; 4 Anhui Medical University, Hefei, 230032, PR China; 5 Southern Medical University, Guangzhou, 510515, PR China; 6 Beijing Tiantan Hospital, Beijing, 100050, PR China; 7 National Institute of Biological Science, Beijing, 102206, PR China; 8 University of Michigan, Comprehensive Cancer Center, Ann Arbor, Michigan, 48109, United States f of America; St Michael's Hospital, University of Toronto, CANADA

## Abstract

Induced neural stem cells (iNSCs) can be directly transdifferentiated from somatic cells. One potential clinical application of the iNSCs is for nerve regeneration. However, it is unknown whether iNSCs function in disease models. We produced transdifferentiated iNSCs by conditional overexpressing *Oct4*, *Sox2*, *Klf4*, *c-Myc*in mouse embryonic fibroblasts. They expanded readily in vitro and expressed NSC mRNA profile and protein markers. These iNSCs differentiated into mature astrocytes, neurons and oligodendrocytes in vitro. Importantly, they reduced lesion size, promoted the recovery of motor and sensory function as well as metabolism status in middle cerebral artery stroke rats. These iNSCs secreted nerve growth factors, which was associated with observed protection of neurons from apoptosis. Furthermore, iNSCs migrated to and passed through the lesion in the cerebral cortex, where Tuj1+ neurons were detected. These findings have revealed the function of transdifferentiated iNSCs in vivo, and thus provide experimental evidence to support the development of personalized regenerative therapy for CNS diseases by using genetically engineered autologous somatic cells.

## Introduction

Neural stem cells (NSCs) promote recovery of neurological disease and show anti-inflammatory, glial scar-inhibitory, and anti-apoptotic effects with neuroprotective functions [1]. These characters make the use of NSCs a promising strategy for the regeneration of damaged brain tissues. However, generation of sufficient numbers of human adult NSCs *in vitro* has remained to be a major limitation for the application of NSCs. With the implication of transdifferentiation reprogramming, however, large quantities of iNSCs can be produced [2,3].

Transdifferentiation is defined as direct reprogramming or conversion of one cell type to another by passing the induction of pluripotent stem cells (iPSCs) [4–6]. This process can be used to reprogram adult somatic cells into either adult stem cells or differentiated cells of another germ layer [7–12]. Classic iPSC transcription factors in conjunction with other neural progenitor cell (NPC)-specific factors or microRNA have been used to transdifferentiate human or mouse somatic cells into iNSCs/iNPCs. [13–17] While transdifferentiated iNSCs can now be derived from human somatic cells, which shows significant potential of cell therapy using autologous grafts, it is not known whether iNSCs/iNPCs can function *in vivo* as NSCs, and few studies have assessed the behavior of iNSCs after transplantation.

In this study, we produced transdifferentiation-induced NSC colonies by direct inducing mouse embryonic fibroblasts (MEFs) with conditional expression of *OSKM* or *Oct4*, *Sox2*, and *Klf4* (*OSK*). These cells were readily expanded *in vitro*. When transplanted into the brains of middle cerebral artery occlusion (MCAO) rats after surgery, the iNSCs significantly improved the motor and sensory functions of the MCAO rats. Importantly, these iNSCs migrated through the lesion region, confirming that they can play an important role in the recovery of MCAO rats.

## Materials and Methods

### Method to get MEF cells

GFP/rtTA, OG2/rtTA MEFs were isolated from E13.5 embryos. OG2/rtTA embryos were generated by crossing of B6; CBA-Tg (Pou5f1-EGFP) 2Mnn/J mice and B6.Cg-Gt (ROSA) 26 Sortm1 (rtTA*M2) Jae/J mice. GFP/rtTA embryos got from crossing of C57BL/6-Tg (CAG-EGFP) 1Osb/J and B6.Cg-Gt (ROSA) 26 Sortm1 (rtTA*M2) Jae/J mice. All of the three transgenic mice strain bought from The Jackson Laboratory.

To get MEF cells, mice at E13.5 gestation were sacrificed by performing cervical dislocation. Saturate mouse abdomen with alcohol, cut back the skin and peritoneal wall with sterile instruments. Then remove uterine horns and place them into sterile, disposable petri dish. Wash uterine horns 3 times with 10ml PBS. Then cut open embryonic sacs and release embryos and place them in fresh dish and wash 3 times with 10ml PBS. Mince tissue with curved dissecting scissors into grain sized pieces for approximately 5–10 minutes. After that, add 2ml Trypsin and mince for an additional few minutes until pieces are further reduced in size, then pipet the cells vigorously up and down. Then place dish into incubator for 20–30 minutes. After removing the minced tissue from incubator, add about 20ml MEF Derivation Culture Media and transfer contents to a sterile 50ml plastic conical tube. Rinse remaining tissue in the plate with a few milliliters MEF Derivation Culture Media (DMEM, 10% FBS and Penicillin-Streptomycin). Transfer media to the 50ml conical tube. Place 10ml MEF Derivation Culture Media into each T75 Flask. Transfer 5ml minced tissue mixture to each flask. Incubate the flasks containing the minced tissue mixture in a 37°C tissue culture incubator overnight. Culture the cells until the flasks are at least 90% confluent.

### Transdifferentiation and iPS reprogramming

TetO-FUW-*Oct4*, TetO-FUW-*Sox2*, TetO-FUW-*Klf4*, TetO-FUW-*cMYC*, or FUW-M2*rtTA* lentivirus supernatants were produced and harvested as described previously [18]. To generate iNSCs, 5×10^4^ fibroblasts in 35 cm^2^ culture dish were infected with lentivirus by different combinations (*OSKM* or *OSK*) in MEF medium. Doxycycline (Sigma–Aldrich, Beijing, China) (2–8 μg/ml) treatment was initiated the following morning (day 0) for 6 days. During the first 3 days of reprogramming, the culture medium was changed to MEF medium with the addition of 1 mg/L Dox. Then it was switched to reprogramming initiation medium (RepM-Ini; knock-out DMEM supplemented with 10% knock-out serum replacer, 5% FBS, 0.1 mM NEAA, 2 mM Glutamax, and 0.055 mM β-mercaptoethanol) for another 3 days also with the addition of Dox. In the final stage, the medium was changed to iNSC medium: DMEM/F12 and neurobasal medium mixed at 1:1, supplemented with 0.05% BSA, 1×B27, 2 mM Glutamax, 20 ng/mL fibroblast growth factor bFGF, and 20 ng/mL epidermal growth factor (EGF). All media were replenished once every other day.

The same batch of viruses was used to induce iPSCs. For iPS reprogramming, 12 day-induction was needed with the addition of Dox. Then the medium was changed to iPSC medium with LIF and 15% FBS. All reagents were purchased from Invitrogen unless otherwise specified.

### Derivation of NSCs from embryonic brain

Timed pregnant wild-type C57 mice were sacrificed on day 14 post-conception. Embryos were aseptically removed and placed into sterile PBS. The meninges were removed under dissecting microscope and pallial or subpallial tissue parts were cut into small pieces. Brain tissue pieces were mechanically disintegrated by triturating with a fire-polish transfer pipette in DMEM/F12 (Invitrogen). 0.05% Typsin-EDTA was used to digest the tissues for 5 min at 37°C. Then 1ml DMEM/F12 was added to the digestion medium and centrifuge for 5min at 1000 rpm. Discard supernatant and resuspended the cells with fresh neural stem cell culture medium (consisted by DMEM/F12, B27, EGF and bFGF), then cultured the NSCs in incubator at 37°C.

### Differentiation of iNSCs and NSCs in vitro

For neuronal differentiation of iNSCs and NSCs, 5×10^4^ cells were plated onto polyornithine- and laminin-coated 24-well plates (Sigma–Aldrich), cultured in DMEM/F12 (1:1), B27, EGF, and bFGF for 2 days, and differentiated in B27 and DMEM/F12 (1:1) for 12 days. For electrophysiological analysis, cells were cultured for an additional 18 days in N2, B27 medium supplemented with 1% serum. For astrocytic differentiation, 5×10^4^ cells were split onto polyornithine-coated 24-well plates and cultured in B27, DMEM/F12 medium plus 5% FBS without FGF and EGF for 5–8 days. For oligodendrocyte differentiation, 5×10^4^ cells were plated onto polyornithine- and laminin-coated 24-well plates and cultured in 10 ng/mL bFGF, 10 ng/mL platelet-derived growth factor (PDGF; Peprotech, Beijing, China), and 10 nM forskolin (Sigma–Aldrich) for 5 days, and then in 200 nM ascorbic acid (Sigma–Aldrich) and 30 ng/mL T3 (Sigma–Aldrich) for 5 days.

### Immunofluorescence

The following antibodies were used: mouse anti-Tuj1 (1:50, Millipore), mouse anti-microtubule-associated protein (MAP) 2 (1:200; Sigma–Aldrich), rabbit anti-GFAP (1:200; Millipore), mouse anti-O4 (1:100, Millipore), mouse anti-CNPase (1:100, Millipore), goat anti-Sox2 (1:100, Santa Cruz), mouse anti-Nestin (1:100; Developmental Studies Hybridoma Bank, Beijing, China), mouse anti-Pax6 (1:100; Developmental Studies Hybridoma Bank), donkey Alexa-555 anti-mouse (1:1000; Invitrogen), and donkey Alexa-555 anti-rabbit (1:1000; Invitrogen). Nuclei were stained with 4',6-diamidino-2-phenylindole (DAPI). The slide-mounted, stained samples were observed using an LSM 510 META confocal microscope (Zeiss, Oberkochen, Germany) with excitation wavelengths of 543, 488, and 405 nm. The channel signals were collected sequentially. Collected images were assembled using Adobe Photoshop (Adobe Systems, San Jose, CA).

### Quantitative Realtime PCR (qRT-PCR) and primers

RNA was isolated using the Trizol reagent (Qiagen, Beijing, China), and cDNA was synthesized using the SuperScript First-strand Synthesis System (Invitrogen). Real-time PCR was performed using the 7900 fast Real-Time PCR System (Applied Biosystems, Beijing, China). Amplification was performed with SYBR Green dye (Takara, Beijing, China). Expression levels were calculated relative to that of glyceraldehyde 3-phosphate dehydrogenase (GAPDH). Primer sequences were previously described by Lujan. [17]

### Teratoma formation test

6 immunodeficient BALB/c nude mice were used to teratoma formation test. Aliquots of 2×10^5^ iNSC1 cells (n = 3) or iNSC11 cells (n = 3) in 0.1 mL PBS were injected subcutaneously into the groin of one side, equal number of GFP/rtTA iPSCs or R1 ESCs were injected subcutaneously into the groin or armpit in the other side of the same mouse as positive control. Animal health was observed twice every week, and diameters of tumors were measured twice every week. Tumor formation was assessed on day 45.

### Animals

Male Sprague–Dawley (SD) rats weighed 270–300 g (Vital River, Beijing, China) were used. Animals were housed under a natural light–dark cycle and allowed access to food and water *ad libitum*.

### Ethics Statement

Animals' care and operation was in accordance with the guidelines approved by the Neurosurgery Institute of the General Hospital of Beijing Military Region, P.L.A. The protocol was approved by the Committee on the Ethics of Animal Experiments of the General Hospital of Beijing Military Region, P.L.A (Permit Number: 2014–044).

### Preparation of MCAO stroke models

Rats were anesthetized with3.6% chloral hydrate (1 mL/100 g, intraperitoneal [i.p.] injection). Permanent middle cerebral artery occlusion was induced using an intraluminal vascular occlusion method based on the technique described by Longa *et al* [19].The right common carotid artery, external carotid artery, and internal carotid artery were exposed. A length of MCAO monofilaments (Beijing Sunbio Biotech company, Beijing, China, 0.26 mm in diameter) was inserted from 18.5 to 19.5 mm, depending on the weight of the animal, and advanced from the external carotid artery into the lumen of the internal carotid artery until it blocked the origin of the middle carotid artery. After surgery, the animals put on a heating pad on the surgical table. The incision region was disinfected with povidone-iodine solution. Each animal also received 1.0 ml of normal saline subcutaneously for volume replenishment after the surgery.

### Cell transplantation

Cells were transplanted under anesthesia 2 days post-MCAO surgery when rats were in the acute phase and the lesion was not stable. Rats were administered two deposits of suspended cells (1×10^6^ cells/10 μL) along the anterior–posterior axis into the cortex at the following coordinates: (i) anterior–posterior (A–P), 1; medial–lateral (M–L), –1.2 mm; dorsal–ventral (D–V), –2.7 mm; (ii) A–P, –0.26 mm; M–L, –1.2 mm; D–V, –2.6 mm. All parameters relate to lambda. Deposits were delivered at 0.5 μL/min using an infusion pump. The needle was left *in situ* for 2 min post-injection before being removed slowly.

### Behavioral testing

All animals underwent behavioral testing before surgery, and 2, 4, 7, and 10 days and 2 weeks post-MCAO by two investigators blinded to the experimental groups. A modified neurological severity score (mNSS) was used to grade various aspects of neurological function. mNSS is a composite of the motor (muscle status and abnormal movement), sensory (visual, tactile, and proprioceptive), and reflex tests (S1 Table). To measure the defect in forelimb somatosensory, small, adhesive-backed paper dots (113.1 mm^2^) were applied as bilateral tactile stimuli to the radial aspect of the wrist of the left forelimb. The time of the rat contacted and removed the stimuli were recorded.

### Magnetic resonance imaging (MRI) and magnetic resonance spectroscopy imaging (MRSI)

MRI was conducted to rats after behavioral tests at Tiantan Hospital, Beijing, China (affiliated with Capital Medical University, Beijing, China). Rats were anesthetized with 10% chloral hydrate (0.3mL/100g, i.p.) and then placed in the MRI scanner (Bruker Pharma Scan 7.0T, 16 cm). Localizer, T2-weighted (T2W), and diffusion-weighted imaging (DWI) were conducted using spin-echo sequences. T2W was used to map the lesion and hemispheric volumes. Twenty contiguous coronal slices (thickness, 1 mm) were acquired (TR = 3040 ms, TE = 37 ms, FOV = 40×40 cm). MRS spectra were obtained with TE = 135 ms and TR = 1500 ms. After MRS analysis, the whole brain was separated into 48 regions, and the relative concentrations of the metabolites NAA, Cho, and Cr were determined in each region. For the regions of interest (ROIs), the value of m was analyzed using the following equation: m = (NAA–Cho)/Cr. In addition, the M value reflects the change in metabolism status of the right brain compared to the contralateral brain. M = mi–mc, where mi and mc indicate the m values of the ipsilateral and contralateral sides, respectively. The mean Mc of all the regions in the right brain reflects the changes in metabolism between different time points.

### Determination of cell secreted factors

Aliquots of about 10^6^ cells were cultured and the supernatants were collected after 24 h in culture. The concentrations of secretory components were determined with ELISA assay kits (Biolab, Beijing, China) and the data was obtained by a Glomax 96 Microplate Luminometer (Promega, Beijing, China).

### Tissue Processing

After anesthesia with 3.5% Chloral hydrate in PBS, animals were perfused transcardially with 100 mL cold saline and 100 ml 4% paraformaldehyde in 0.1 mol/L PBS. For frozen sections, after post fixation overnight, brains were cryoprotected in 30% sucrose for 24 h, and then frozen in OCT at –80°C.

### Electron microscopy scanning

For electron microscopy scanning, three small tissue blocks about 1 mm^3^ in the ischemic penumbra around the region of ischemic necrosis in the right brain were taken and fixed in 2% osmic acid solution. After immersion in 0.2 M phosphate buffer for 10 min×3, the samples were fixed in 2% osmic acid solution at 4°C until embedding. Ultrathin sections were 0.5 mm^2^and100 nm thick. All of the microscopy images were obtained by a H7650 microscopy (Hitachi, China).

### Statistical analysis

All data in this study were presented as means ±SD. Error bars in the figures mean SD except where indicated otherwise. Two-way ANOVA tests were conducted that examined the effect of day and treatment on mNSS score and adhesive removal time. For a nonparametric comparison of MRS values between PBS and iNSC groups, we used Mann–Whitney U-test. SPSS was used to conduct the statistical tests and Prism 5 was used to conduct the image. The value of <0.05 was considered to be significant.

## Results

### 1. Derivation of iNSC colonies through direct reprogramming by conditional overexpression of OSKM or OSK

**Fig 1:**
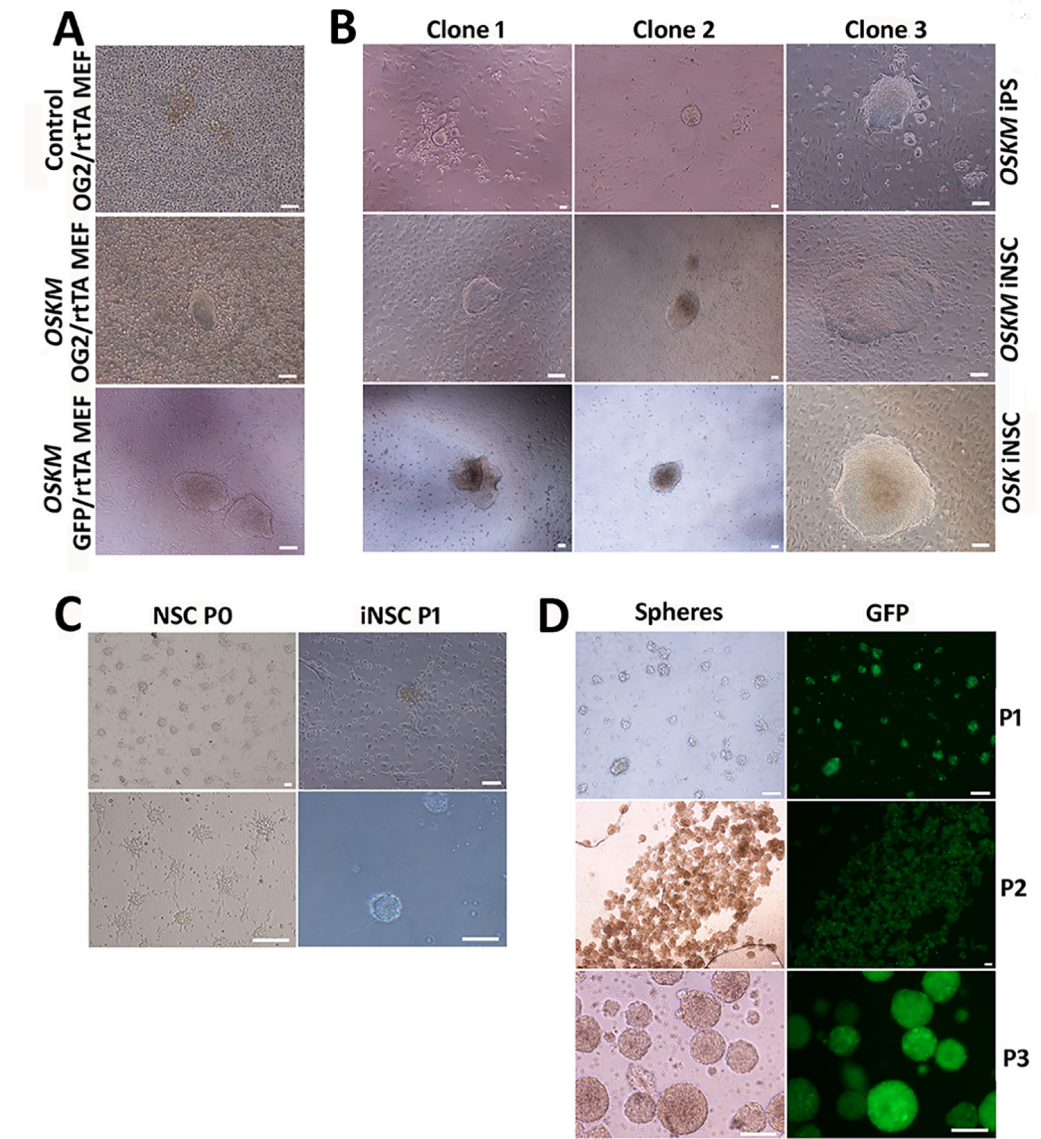
iNSCs directly reprogrammed from MEF cells by conditional induction of classic iPS transcription factors *OSKM* or *OSK*. (A) OG2/rtTA MEF or GFP/rtTA MEF (4×10^5^) cells were transfected with a tetracycline (*tet*)-on controlled *OSKM* lentivirus and induced for 6 days by doxycycline. iNSC colonies are shown onday 13. (B) The morphology of GFP/rtTA MEF-derived *OSKM*-induced iPS clones, *OSKM*-induced iNSC clones, and *OSK*-induced iNSC clones. (C) Comparison of iNSC neurospheres with those derived from primary NSCs from the adherent brain tissues. (D) Expansion of GFP/rtTA iNSCs and the expression of GFP *in vitro*. Scale bar = 100 μm.

Kim *et al*. reported that overexpression of *OSKM* for 6 days was sufficient to generate Pax6^+^ colonies, and constitutive overexpression of *OSKM* without precise temporal control may be detrimental. Therefore, we used a tetracycline (*tet*)-on system with the addition of doxycycline (Dox) in culture medium to control the conditional overexpression of *OSKM* or *OSK* in OG2/rtTA MEF or GFP/rtTA MEF for only 6 days to generate directly reprogrammed NSCs. Colonies in iNSC medium appeared in 13–26 days of induction (Fig 1A). We also induced the overexpression of *OSKM* for 12 days to obtain GFP/rtTA iPSCs in iPSC culture medium with the addition of LIF and serum. Morphologically, the *OSKM* iNSC clones were similar to epistem cells, while *OSK* iNSC clones were more similar to iPSC or ESC (Fig 1B).

Individual colonies were transferred into 96-well plates and digested with accutase into single cells, which were then cultured in iNSC medium for expansion. Neurosphere-like colonies expressing the GFP marker inherited from GFP/rtTA MEFs were obtained from dispersed cells and exhibited a rosette-like appearance, which was similar to the process of deriving NSC spheres from the mouse brain (Fig 1C). They became detached from the dish as they enlarged and were suspended in culture medium as spheres (Fig 1D). At the first passage (P1), the culture medium was collected and centrifuged to separate neurospheres for digestive passage. We repeated the transdifferentiation process three times and established fifteen *OSKM*-induced and three *OSK*-induced self-renewing NSC lines. Some lines had been expanded up to P50.

### 2. Transdifferentiated NSC lines show specific NSC gene expression profiles

**Fig 2:**
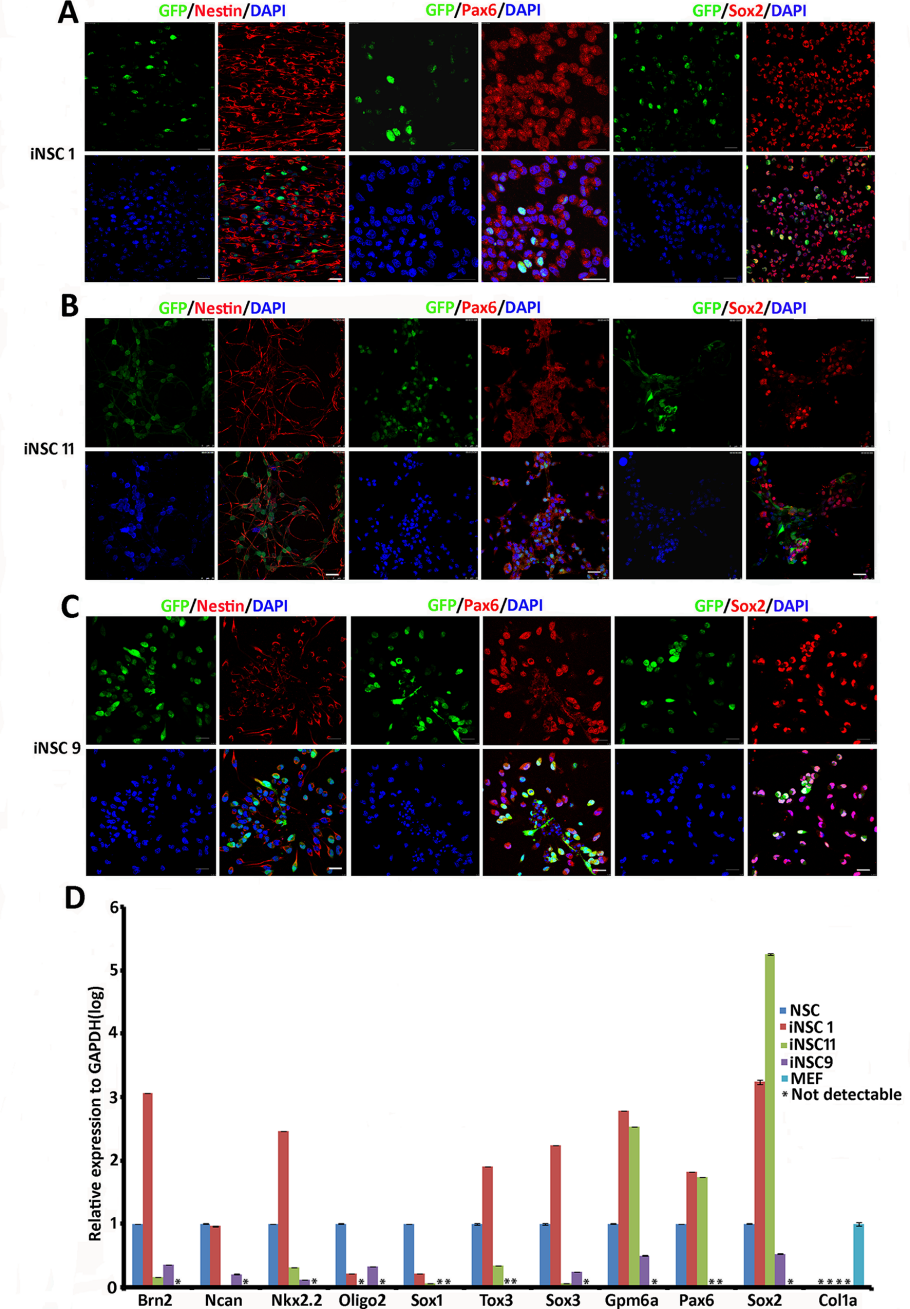
Immunostaining and mRNA expression profiling of iNSC lines for NSC markers. Expression of NSC markers Nestin, Pax6, and Sox2 in (A) *OSKM-*induced iNSC1 cells (P19), (B) *OSKM-*induced iNSC11 cells (P19), and in (C) *OSK-*induced iNSC9 cells (P11). Scale bar = 25 μm. (D) qRT-PCR analysis of neural progenitor specific genes expressed by *OSKM-*induced cell lines iNSC1 and iNSC11, and *OSK-*induced iNSC9 cells *vs*. those of NSCs derived from the mouse brain.

To determine whether the established cell lines are NSC lines, we performed molecular characterization at the protein and mRNA level. Immunostaining of *OSKM*-induced iNSC1 (P19), iNSC11 (P19), and *OSK-*induced iNSC9 (P11) cells resulted in the detection of Nestin, Pax6, and Sox2, characteristic markers of NSCs, in all these three transdifferentiated cell lines (Fig 2A, 2B and 2C).

To determine whether these transdifferentiated cell lines exhibited a transcriptional profile similar to NSCs from the mouse brain, we compared the mRNA levels by qRT-PCR (Fig 2D). NSCs derived from the brains of E14 C57 animals were used as positive controls and MEFs derived from E13.5 C57 animals were used as negative controls. *OSKM*-induced cell lines iNSC1 (P7) and iNSC11 (P7), as well as *OSK-*induced cell line iNSC9 (P11) revealed the gene expression pattern expected for NSC endogenous expression levels of the neural progenitor cell genes. *Brn2*, *Ncan*, *Nkx2*.*2*, *Olig2*, *Sox1*, *Tox3*, *Sox3*, *Gpm6a*, *Pax6*, *Sox2*, and the MEF-specific gene *Col1a* were quantified. The mRNA levels of some of these genes in iNSC1 and iNSC11 were similar to those in NSCs from mouse brains. However, not all iNSC lines exhibited identical miRNA expression patterns, suggesting that different degrees of reprogramming occurred. *OSKM* induced iNSC1 cells exhibited higher expression of the following seven genes than NSCs: *Brn2*, *NKX2*.*2*, *Tox3*, *Sox3*, *Gpm6a*, *Pax6*, and *Sox2*. In contrast, the expression of *Oligo2* and *Sox1* were lower than those in NSCs. *OSK*-induced cells (iNSC9) also expressed NSC-specific genes, but at lower levels. Collectively, these results indicate that iNSC1 cells were reprogrammed to more closely resemble NSCs. In addition, none of the three iNSC lines tested expressed the MEF-specific *Col1a* gene, suggesting that they are distinct from MEF with established NSC gene profiles.

### 3. iNSCs differentiate into mature neurons, astrocytes, and oligodendrocytes in vitro

**Fig 3:**
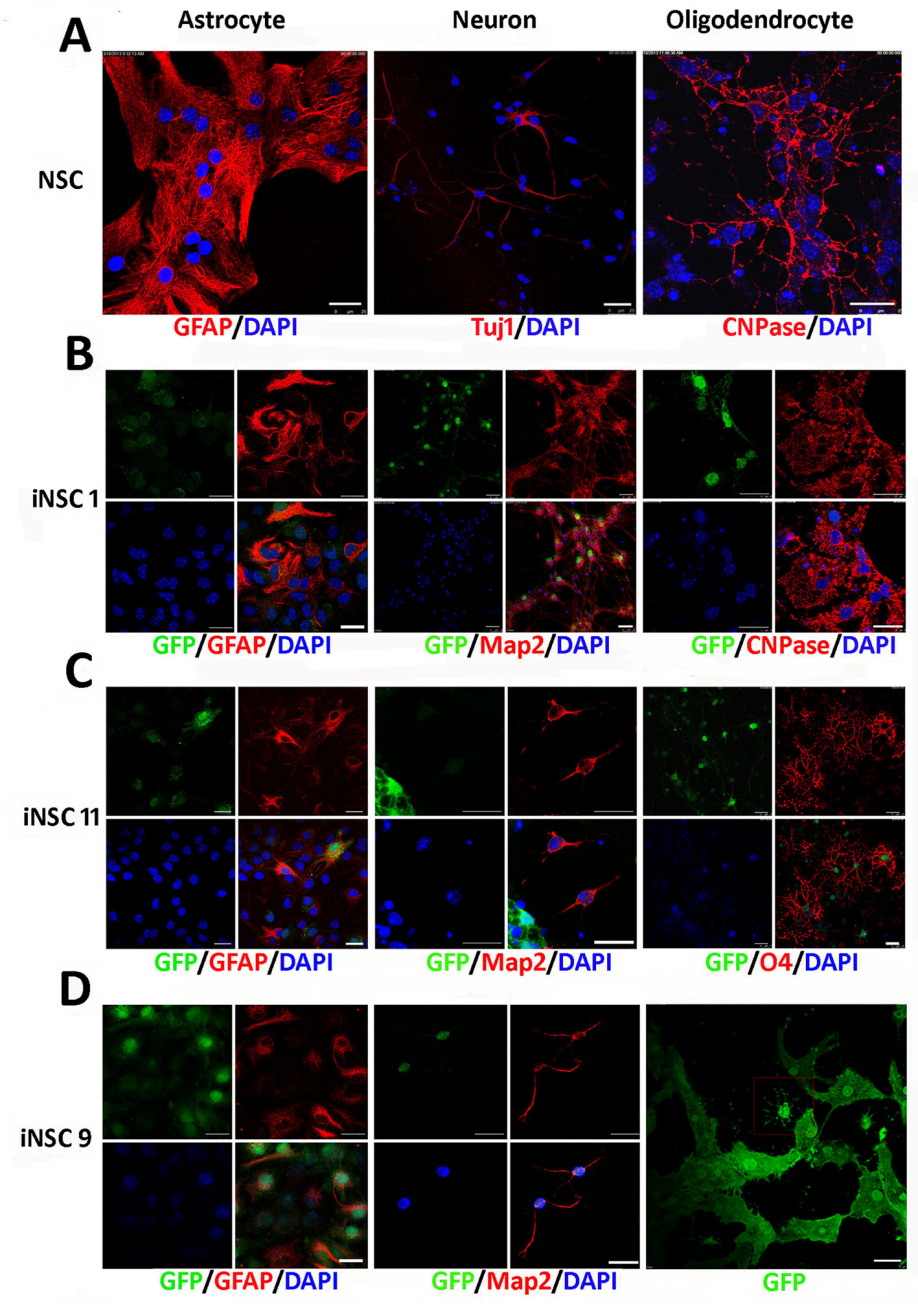
Differentiation of iNSC lines in vitro. GFP was inherited from GFP/rtTA MEFs. Immunostains were performed on differentiated iNSCs with antibodies against the indicated markers: GFAP (astrocyte marker); TUJ1 (pan-neuronal marker); MAP2 (mature neuronal marker); O4 and CNPase (oligodendrocyte markers). (A) Differentiation of NSCs derived from the mouse brain. (B) Differentiation of iNSC1 cells. (C) Differentiation of iNSC11 cells. (D) Differentiation of iNSC9 cells. GFAP^+^ astrocytes, MAP2^+^ neurons, and Oliogodendrocytes-like cells were differentiated. Scale bar = 25μm.

NSCs derived from the mouse brain can be differentiated into astrocytes, neurons, and oligodendrocytes (Fig 3A). To determine whether iNSCs have a differentiation potential similar to that of NSCs derived from E14 mice as shown in Fig 3A, we used cell type-specific methods to differentiate iNSCs into astrocytes, neurons, and oligodendrocytes (Fig 3B, 3C and 3D). Addition of 5% FBS induced differentiation of all three iNSC lines into astrocytes, as indicated by staining for GFAP three days after differentiation. iNSC1 (P11), iNSC11 (P7), and iNSC9 (P9) were induced to differentiate into neurons that were positive for MAP2 when EGF and bFGF were removed from the media for 12 days, while NSC cell-derived neurons were positive for Tuj1. To assess the function of mature neurons, these differentiated iNSCs were further cultured for 30 days. More than 90% of *OSKM*-induced iNSC1 cells differentiated into neurons. In addition, we used a two-step method to differentiate iNSCs into oligodendrocytes. *OSKM-*induced iNSC1 and iNSC11 differentiated into oligodendrocytes, as evidenced by positive immunostaining with an antibody against CNPase or O4, while *OSK*-induced iNSC9 differentiated into oligodendrocyte-like cells. Together, these results indicate that iNSCs have neural progenitor multipotency.

### 4. iNSC-derived tumors are absent from immunodeficient nude mice

**Fig 4:**
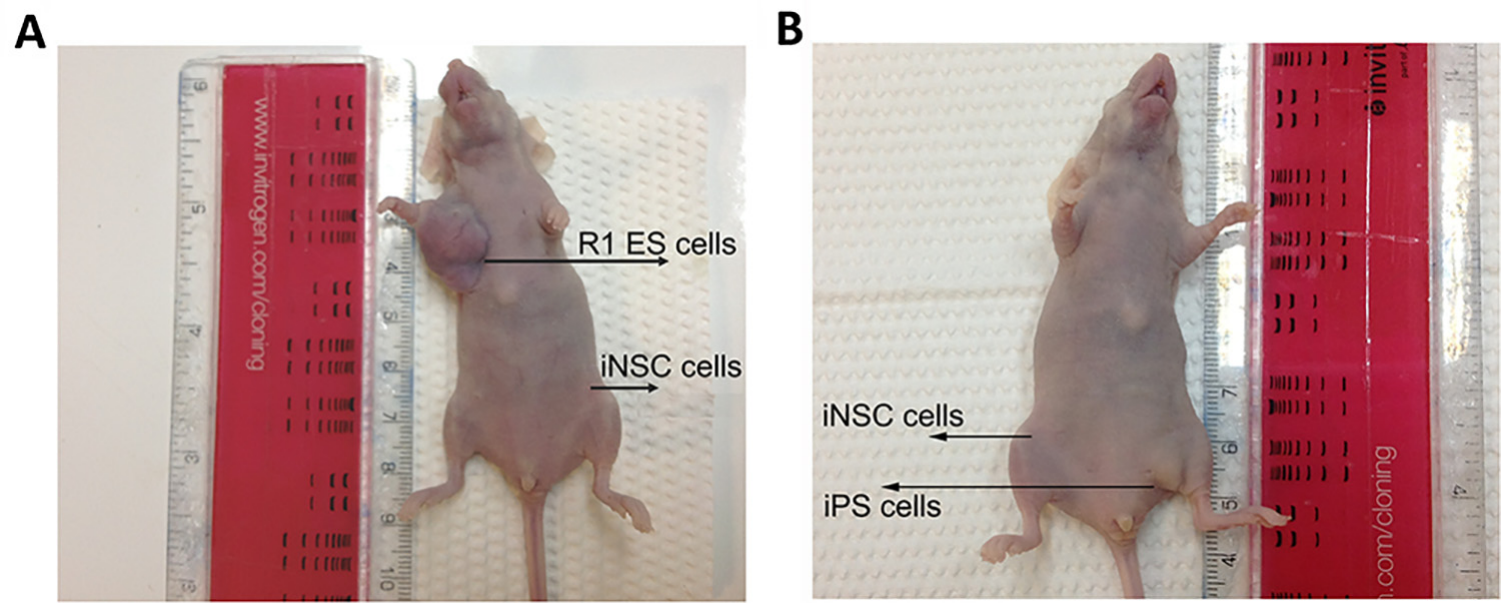
Tumor risk determination of iNSCs. (A) 2×10^5^ iNSC1 cells or ESCs were injected into the subcutaneous region of the same immunodeficient BALB/c nude mouse (n = 3) and tumor formation was assessed on day 45. Tumors were found in ESCs injected sites, no tumor were found in iNSC1 injected sites. (B) Tumor formation analysis of 2×10^5^ iNSC11 cells and GFP/rtTA iPSCs in the same immunodeficient BALB/c nude mouse on day 45. Tumors were observed in iPSCs injected sites but not in iNSC11 injected sites.

The risk of tumor formation has significantly limited the use of iPSC. The iNSCs exhibited both pluripotency and differentiation ability as shown above; we then investigated their tumor formation potential *in vivo*. Aliquots of 2×10^5^ iNSCs, R1 ESCs and GFP/rtTA iPSC were injected subcutaneously into the groin of immunodeficient BALB/c nude mice. Teratomas were observed at D45 (diameters from 0.5–1.5cm) at the sites of ESCs and iPSCs injection but not at the sites injected with the iNSCs (Fig 4A and 4B). And no signs of pain and distress in the animals during the tumor growth period were observed.

Furthermore, to determine whether the neurospheres could form tumor, iNSCs were collected and half of the cells were digested into single cells and suspended in 0.1mL PBS, while the other half were suspended directly in 0.1 mL PBS as neurospheres. Either single cells or neurospheres (containing ~2×10^5^ cells) were injected subcutaneously into the groin region of immunodeficient nude mice. Equal numbers of R1 ES cells were injected into the same mouse as a positive control. No tumors were evident 45 days after the injection of iNSC1 cells or 55 days after injection of iNSC11 cells. In contrast, teratoma arose at the injection sites of ES cells (data not shown). These observations indicate that iNSCs, either as single cells or as spheres, do not form tumors *in vivo*.

### 5. Transplantation of the iNSCs in vivo promotes functional recovery of MCAO rats and endorses the recovery of metabolism status

**Fig 5:**
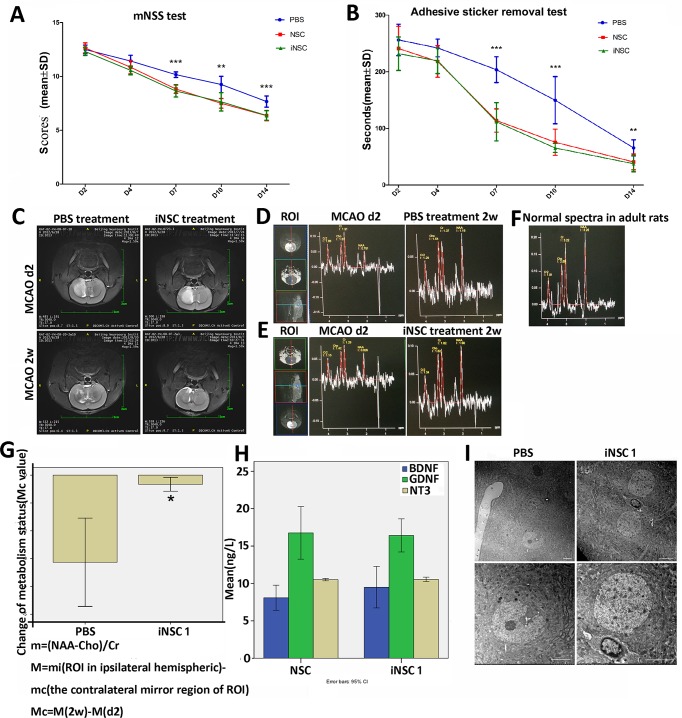
Behavior tests, MRI and MRS and neuron protection mechanism analysis of MCAO rats treated with iNSCs. (A) mNSS test of MCAO rats that received PBS(n = 12) or NSCs (n = 12) or iNSC1 (n = 12) treatment post MCAO surgery. NSCs or iNSCs treatment was significantly less mNSS score than PBS treatment on days 7, 10, 14 after transplantation (p <0.05). (B) Adhesive sticker removal test. NSCs or iNSCs treatment was significantly less adhesive removal time than PBS treatment on days 7, 10, 14 after transplantation (p <0.05). (C) T2 MRI of rats with identical lesion size in the same region of the right brain 2 days before treatment and 2 weeks post-MCAO after PBS or iNSC1 cell treatment. (D) MRS images of the MCAO rat treated with PBS or (E) with iNSCs in the ROI2 days and 2 weeks post-MCAO. (F) Magnetic resonance spectroscopy (MRS) images of normal adult rat. (G) Mean value of Mc represents changes of metabolic status in the right brain ROIs of MCAO rats treated with PBS (n = 12 in 3 rats) or iNSC (n = 8 in 2 rats), *P<0.05. The error bars are SE. NAA, N-Acetylaspartate. Cr, Creatine. Cho, Choline. (H) Elisa tests of nerve growth factors BDNF, GDNF, and NT3 secreted by iNSC1 cells and NSCs derived from the E14 of C57 mouse brain. (I) Comparation of neurons in the penumbra of the rats received PBS or iNSCs treatment 8 weeks post-MCAO. Scale bar = 5μm.

To examine the function of iNSCs *in vivo*, a total of 65 rats of similar body weight underwent permanent MCAO surgery. Survived rats were separated to three groups at day 2.The negative control group was injected with PBS (*n* = 12). The positive control group was injected with 10^6^ NSC cells (*n* = 12), and the experimental group received 10^6^ iNSCs (*n* = 12). Neurological deficits were assessed using the mNSS test (Fig 5A) and adhesive sticker removal test (Fig 5B). After MCAO, all of the animals developed marked neurological deficits, and there were no differences among the groups prior to transplantation. Neurological performance was monitored until day 14 post-transplantation. Compared to PBS-treated controls, the iNSC- and NSC-transplanted groups showed similar progressively enhanced recovery of motor skills in the mNSS test (Fig 5A). A two-way ANOVA was conducted that examined the effect of day and treatment on mNSS score. There was a statistically significant interaction between the effects of day and treatment on mNSS score, F = 3.867, p = 0.001. Simple main effects analysis showed that NSCs or iNSCs treatment was significantly less mNSS score than PBS treatment on days 7, 10, 14 after transplantation (p <0.05). Also a two-way ANOVA was conducted that examined the effect of day and treatment on adhesive removal time (Fig 5B). There was a statistically significant interaction between the effects of day and treatment on adhesive removal time, F = 4.214, p = 0.000. Simple main effects analysis showed that NSCs or iNSCs treatment was significantly less adhesive removal time than PBS treatment on days 7, 10, 14 after transplantation (p <0.05). These observations reveal that iNSCs promote the recovery of MCAO rats to a similar extent as NSCs.

To see if iNSCs can reduce the lesion size, MRI analyses of the hemispheric area were performed and showed a clear image of the lesion hemisphere on the right side in all MCAO rats. Two rats had been identified with almost identical lesion volume in the same region by hyperintensity on T2 weighted images two days post-MCAO. Using these two rats, one received iNSC treatment and the other received PBS. The animal treated with iNSC showed much better recovery with smaller lesion size than that treated with PBS 2 weeks post-MCAO (Fig 5C and S2 Fig).

Metabolite changes in the brain often precede structural changes, and magnetic resonance spectroscopy (MRS) can demonstrate these changes before MRI [20]. The rats carried a long TE (TE = 135) sequence to obtain MRS images in a 7.0T MR scanner [21]. Two MCAO rats with almost the same lesion size on day 2 were chosen and their MRS maps are shown in Fig 5C and 5D. After MRI, the animals received PBS (Fig 5D) or 10^6^ iNSCs (Fig 5E). The same ROIs were chosen near the injection site of iNSCs or PBS. Two weeks post-MCAO, both animals were subjected to MRI with the same sequence. All scans were performed with the same sequence and the same parameters and the normal spectra in adult rats were shown in Fig 5F. As shown in Fig 5C and 5D, the rat received iNSC treatment demonstrated better recovery than PBS treatment when compared with normal MRS image of adult rats.

N-Acetylaspartate (NAA) is a metabolite in the brain that can be used as a marker of neuronal and axonal density and viability. Absenceor reduced concentration of NAA correlates with neuronal degradation or loss. Creatine (Cr) is a marker of intracellular metabolism and energetic systems. Choline (Cho) is a marker of cell proliferation. Ratios of these metabolites NAA/Cho, Cho/Cr, NAA/Cr, (Cho+Cr)/NAA, NAA/(Cho+Cr) can be used to reflect the metabolic status of brain tissues. [22–25] NAA/Cr was reported to decrease and Cho/Cr was reported to increase in the ischemic brain after stroke. [26] Here we chose the m ratio calculated as (NAA-Cho)/Cr which combines three markers to reflect the metabolic status of MCAO brains. Furthermore, the brains were separated into small ROIs, and the corresponding M values in each ROIs of the right brain were analyzed and corrected according to the mirror region of the left brain. Two weeks post-MCAO, the mean Mc values in the ROIs of the right brain were determined. The iNSC group ROIs (n = 12 in 3 rats) showed significantly (*P*<0.05) better metabolic status change than the PBS group ROIs (n = 8 in 2 rats) (Fig 5G)

NSCs have the ability to secrete many growth factors or neurotrophins which can maintain or promote the growth of neuronal cells [27, 28], also can help to stimulate and control neurogenesis which is a process to grow new neurons from neural stem cells. Among them, BDNF, GDNF and NT-3 are high conservative in function and sequence of mouse, rat and human. BDNF (Brain-derived neurotrophic factor) can support the survival of existing neurons, and encourage the growth and differentiation of new neurons and synapses [29]. GDNF (glial-cell-line-derived neurotrophic factor) can enhance survival of mammalian dopaminergic neurons, rescue developing motor neurons from natural programmed cell death in vivo, promote the survival of motor neurons [30]. NT-3 (Neurotrophin-3) helps to support the survival and differentiation of existing neurons, and encourages the growth and differentiation of new neurons and synapses [31]. ELISAs were carried out to determine whether our iNSCs have such capability. The culture media of iNSC1 (P39, P41, P42) and NSC (P29, P31, P32) were collected, and the concentrations of BDNF, GDNF, and NT-3 were determined. As shown in Fig 5H, iNSCs demonstrated almost the same capability to secrete these nerve growth factors compared with NSCs. Of note, these growth factors may be associated with the treatment using iNSCs (Fig 5A, 5B, 5C, 5E and 5G), which may significantly promoted the recovery of the MCAO rats in acute stage of ischemic stroke.

The penumbra is a ischemic region around the ischemic center, which can be repaired after ischemic stroke, and is critical to the recovery [32]. We proceeded by performing the following experiments to see if the penumbra treated with iNSCs recovers better than control (PBS treatment). Eight weeks post-MCAO and treatment, the tissues in the ischemic penumbra were observed under the electron microscopy (Fig 5I). In the PBS-treated brain, most of the neurons had obvious nuclear membrane invagination and the number of second lysosomes was higher, representing direct evidence of apoptosis. In iNSC-treated brains, neurons showed intact nuclear membrane; no secondary lysosomes, and appeared healthy, compared with the adjacent apoptotic neurons. These results suggest that iNSCs could protect the neurons in the penumbra region from apoptosis *in vivo*.

### 6. Survival and migration of iNSCs along the ischemic cortex lesion and differentiation of iNSCs in the MCAO rat brain

**Fig 6:**
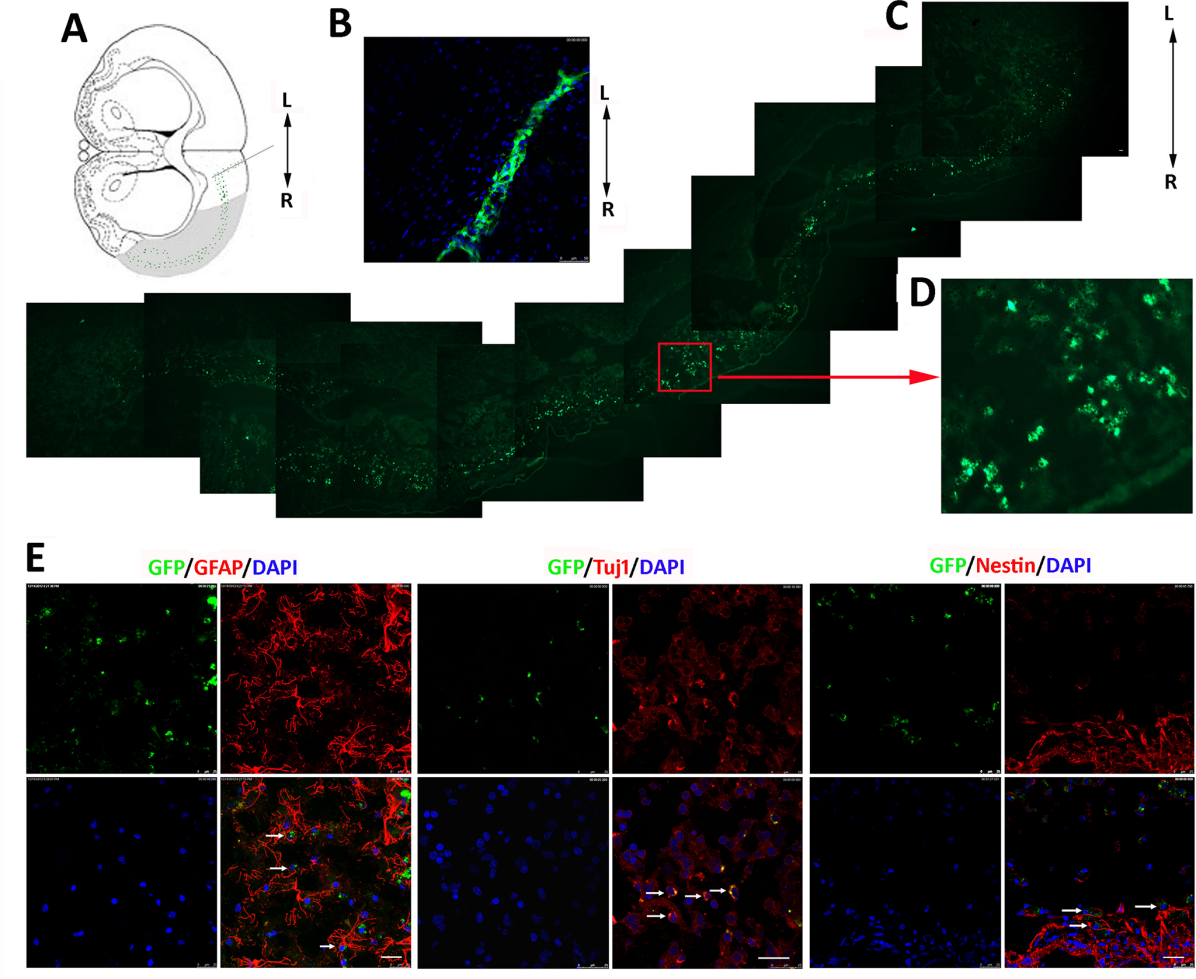
Migration and differentiation of iNSC-derived cells in MCAO rats. (A) Diagram showing the sites on the A–P axis at which iNSCs were transplanted and the migration pathway of the transplanted iNSCs (green dots) in the lesion (shaded area). (B) One day post-transplantation, iNSCs with GFP had assembled at the injection site. (C) Migrated graft cells expressing the GFP marker across the lesion of MCAO rats 6 weeks after transplantation. (D) Higher magnification of demarked area in C. Migrated cells survived in the lesion of the right cerebral cortex. Scale bar = 50 μm. (E) Confocal images: Graft cells labeled with GFP are green, nuclei stained with DAPI are blue, and various phenotypic markers are in red. iNSC1 cells differentiated into GFAP-positive astrocytes in 2 weeks, and Tuj1-positive neurons in 6 weeks. Undifferentiated iNSCs were detected by Nestin antibody. Scale bar = 25 μm.

Although iNSCs have demonstrated the ability to improve the microenvirment, it is not clear if they can survive for a long time and if they can migrate to the lesion. In addition, it is not clear if they can differentiate *in vivo* to replace died neuronal cells. In the following experiments, iNSCs were transplanted into the non-ischemic tissue adjacent to the lesion in the right hemisphere (Fig 6A). One day post-transplantation, iNSCs had assembled at the injection site (Fig 6B). Six weeks post-transplantation, the majority of the grafted cells had migrated along and passed through the ischemic cortex lesion (Fig 6C). The enlarged image indicates that the cells survived and retained their morphology, exhibiting a clear nucleus (Fig 6D). Some cells reached the ipsilateral ventricles and corpus callosum; these could be tracked by detecting the GFP marker. The observed migration of iNSCs in the lesion confirms that they play an important role in the functional recovery of the MCAO rats.

We also examined the differentiation of the transplanted iNSCs *in vivo* (Fig 6E and S1 Movie). iNSCs differentiated into astrocytes 2 weeks after transplantation, as indicated by GFAP labeling. Neurons were detected using the neuronal marker Tuj1 antibody in the migrated cells 6 weeks after cell transplantation. In addition, after 8 weeks, a proportion of GFP-expressing cells were positive for the NSC marker Nestin.

## Discussion

The central nervous system (CNS) cannot regenerate itself by nature. For neural tissue damage repair, neural stem cell implantation offers a promising solution [33]. Exogenous NSC implantation has been shown to promote functional recovery of CNS diseases [34–36]. However, NSCs cannot be obtained easily in large numbers *in vitro*. Alternatively, reprogrammed iPSCs can help get enough neuronal progenitor cells *in vitro* and have been proved to be functional *in vivo*. Human iPSC-derived cortical neuronal progenitors differentiated into neurons, which integrate in stroke-injured cortex and improve functional recovery [37]. Human iPSC-derived oligodendrocyte progenitor cells can myelinate and rescue congenital hypomyelination in mouse model [38]. Transplanted autologous iPSC-derived neural progenitors survive for up to 6 months and differentiate into mature neurons in the brains of MPTP-induced PD rhesus monkey [39].In order to get the above progenitors, two key processes are needed: one is the induction of iPSC, the other is their differentiation into the targeted cells. These processes are complicate and take a long time to complete before personal treatment. In addition, iPSCs can form tumor in the post-ischemic brain, making the application of these cells risky. [40]

Transdifferentiation is a novel strategy of reprogramming to obtain iNSCs bypassing the iPSC stage. There has been remarkable progress in the development of methods for obtaining animal and human iNSCs by transdifferentiation. However, to date, there are few reports regarding the functions of transdifferentiation-induced NSCs *in vivo*. In this report, our data demonstrate that NSCs can be induced directly from mouse somatic cells by accurate overexpression of the classic iPS transcription factors *OSKM* or *OSK*. This is consistent with Kim’s report, but we get iNSC neurospheres with self-renewal ability which can be expanded *in vitro* and establish cell lines. These iNSC lines have shown the similar expression profile of neural progenitor specific genes [17]. Also they have revealed trilineage differentiation potential to astrocytes, mature neurons with AP and oligodendrocytes *in vitro*. In addition, they did not generate tumors in immunodeficient nude mice, in contrast to ESC or iPSC. Functionally, we found that these iNSCs promoted the recovery of motor ability and sensory ability in MCAO rats, representing evidence of their function *in vivo*. Furthermore, the iNSCs demonstrated similar capability to secrete nerve growth factors as NSCs, which is one of the molecular requirements for their function *in vivo*. Electron microscopy revealed that while the neurons in the brain penumbra were still on the process of apoptosis in a long time after stoke without rescue measures, but iNSCs prevented the neurons in the penumbra of MCAO rats from apoptosis. These *in vivo* data suggest that iNSCs promote recovery from ischemic injury by neuron protection and anti-apoptotic mechanisms [41]. MRI and MRS analyses indicate that iNSCs can improve the metabolism status in the ipsilateral hemisphere of MCAO rats. The lesion size reduction was different between PBS and iNSC-treated rats who had almost the same lesion size at day 2 after MCAO. And the lesion region in iNSC-treated rat was apparently smaller in our model. Of note, no tumor growth was observed in iNSC-transplanted rats.

To acquire effective cell therapy through cell replacement of damaged brain cells by iNSCs in vivo, several critical questions need to be addressed. Firstly, to help targeted differentiation of iNSCs in vivo into mature neuronal cells to be used for different illnesses, primary differentiation to neuron precursor cells or oligodendrocyte precursor cells may need to be done in vitro [42]. Secondly, to improve the therapeutic efficiency of exogenous iNSCs, derived neuron integration into the damaged brain network needs to be driven by the use of some protein-biomaterial materials, either alone or in combination with the neurotrophic factors [43]. Third, to monitor and ensure the correct integration of exogenous iNSC-derived neuronal cells to the cortical network, further studies are warranty. Mini brain which can model human brain development may represent an ideal model to define it in vitro [44].

In addition, to use iNSCs as a medical treatment in clinical, we should aim at using virus-free protocol to get patient specific iNSCs, which can be reached with the development of novel reprogramming technology [45–47]. Furthermore, the optimal time and location to transplant donor iNSC-derived neural cells should be determined to ensure their optimal survival and function in vivo [48]. Last but not the least, the index values of the recovery degree used to evaluate the therapeutic efficacy of transplanted iNSCs, including the ones used in this study, should be normalized to reliably evaluate the function of iNSCs in vivo.

## Conclusion

Transdifferentiated iNSCs can functional *in vivo*. These results reveal promises to treat CNS diseases in clinical by using induced neural stem cells transdifferentiated from autologous somatic cells.

## Supporting Information

S1 Fig RT-PCR detection of Pluripotency genes. (TIF)

S2 Fig Lesion area in PBS or iNSC treated MCAO brains. (TIF)

S1 Movie iNSCs differentiation in the ischemic brain after MCAO. (ZIP)

S1 Table Modified neurological severity score (mNSS). (PDF)
