# Supplementary figures and images for: Transdifferentiation-Induced Neural Stem Cells Promote Recovery of Middle Cerebral Artery Stroke Rats
Source: PLoS One. 2015 Sep 9;10(9):e0137211. doi: 10.1371/journal.pone.0137211 (PMC4564190; doi:10.1371/journal.pone.0137211)

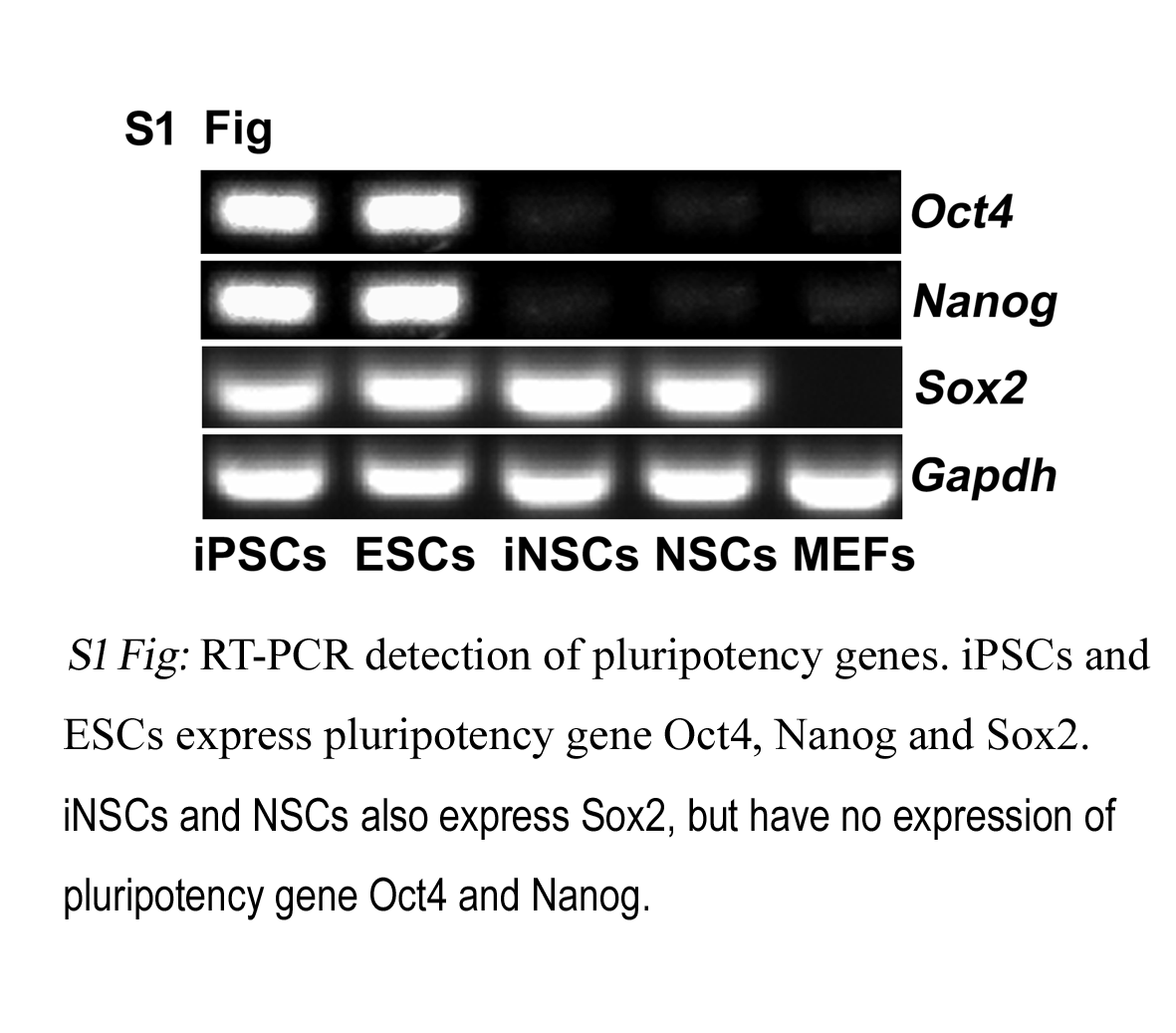

Supplement: S1 Fig — (TIF) [file pone.0137211.s001.tif]

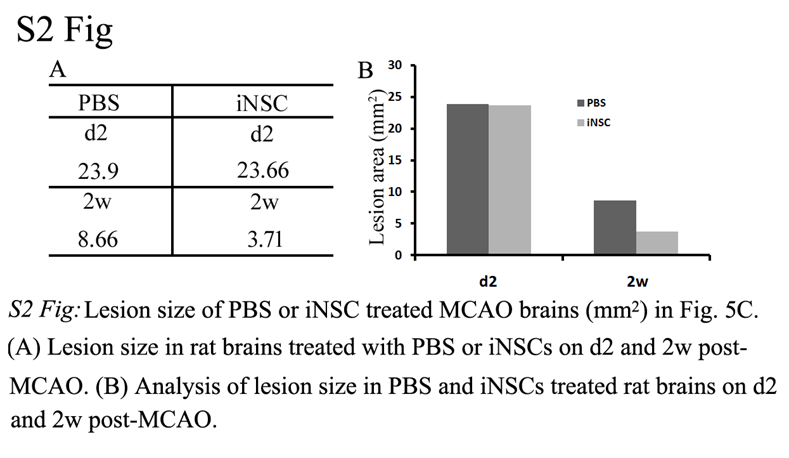

Supplement: S2 Fig — (TIF) [file pone.0137211.s002.tif]
