# Supplementary material for: Transdifferentiation-Induced Neural Stem Cells Promote Recovery of Middle Cerebral Artery Stroke Rats
Source: PLoS One. 2015 Sep 9;10(9):e0137211. doi: 10.1371/journal.pone.0137211 (PMC4564190; doi:10.1371/journal.pone.0137211)
Supplement: S1 Table — (PDF) [file pone.0137211.s004.pdf]

S1 Table: Modified neurological severity score (mNSS)

Tests	Points
**Motor tests**	6
Raising rat by the tail (normal=0; maximum=3)	3
Flexion of forelimb	1
Flexion of hindlimb	1
Head moved >10° to vertical axis within 30 s	1
Placing rat on the floor (normal=0; maximum=3)	3
Normal walk	0
Inability to walk straight	1
Circling toward the paretic side	2
Fall down to the paretic side	3
**Sensory tests**	2
Placing test (visual and tactile test)	1
Proprioceptive test (deep sensation, pushing the paw against the table edge to stimulate limb muscles)	2
**Beam balance tests** (normal=0; maximum=6)	6
Balances with steady posture	0
Grasps side of beam	1
Hugs the beam and one limb falls down from the beam	2
Hugs the beam and two limbs fall down from the beam, or spins on beam (>60 s)	3
Attempts to balance on the beam but falls off (>40 s)	4
Attempts to balance on the beam but falls off (>20 s)	5
Falls off: No attempt to balance or hang on to the beam (<20 s)	6
**Reflexes absent and abnormal movements**	4
Pinna reflex (head shake when touching the auditory meatus)	1
Corneal reflex (eye blink when lightly touching the cornea with cotton)	1
Startle reflex (motor response to a brief noise from snapping a clipboard paper	1
Seizures, myoclonus, myodystony	1
Maximum points	18
